# Comparative analysis of the mitochondrial genome of *Dermacentor steini* from different regions in China

**DOI:** 10.1017/S0031182022001639

**Published:** 2023-02

**Authors:** Huijuan Yang, Ting Chen, Wenge Dong

**Affiliations:** Yunnan Provincial Key Laboratory for Zoonosis Control and Prevention, Institute of Pathogens and Vectors, Dali University, Dali, Yunnan 671000, China

**Keywords:** Comparative analysis, *Dermacentor steini*, Mitochondrial genome, Phylogeny, Tick

## Abstract

Ticks are a group of blood-sucking ectoparasites that play an important role in human health and livestock production development as vectors of zoonotic diseases. The phylogenetic tree of single genes cannot accurately reflect the true kinship between species. Based on the complete mitochondrial genome analysis one can help to elucidate the phylogenetic relationships among species. In this study, the complete mitochondrial genome of *Dermacentor steini* (isolate Longyan) was sequenced and compared with the mitochondrial genes of 3 other Chinese isolates (Nanchang, Jinhua and Yingtan). In *Dermacentor steini* 4 isolates had identical or similar mitochondrial genome lengths and an overall variation of 0.76% between sequences. All nucleotide compositions showed a distinct AT preference. The most common initiation and stop codons were ATG and TAA, respectively. Fewer base mismatches were found in the tRNA gene of *D. steini* (isolate Longyan), and the vicinity of the control region and tRNA gene was a hot rearrangement region of the genus *Dermacentor*. Maximum likelihood trees and Bayesian trees indicate that *D. steini* is most closely related to *Dermacentor auratus*. The results enrich the mitochondrial genomic data of species in the genus *Dermacentor* and provide novel insights for further studies on the phylogeographic classification and molecular evolution of ticks.

## Graphical abstract

**Figure:**
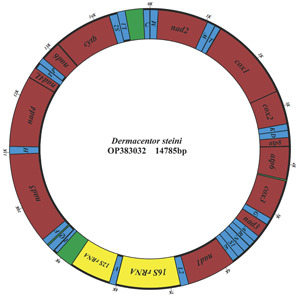

## Introduction

Ticks are a group of blood-sucking ectoparasites that often parasitize on the body surfaces of mammals, birds, reptiles and amphibians (Burnard and Shao, 2019). Ticks have become the second largest vector of infectious diseases worldwide after mosquitoes because they can carry a variety of pathogens such as bacteria, fungi, viruses, nematodes and blood protozoans (Mead *et al*., 2015). Ticks can also cause severe allergic or toxic reactions, temporary paralysis and secondary infections in humans, livestock and other vertebrates through their bites, seriously endangering human health as well as livestock development (Chen *et al*., 2010).

The genus *Dermacentor* (Ixodida: Ixodidae) is a recently evolved genus with relatively limited species diversity compared to other genera (e.g. *Ixodes* and *Haemaphysalis*) in the family Ixodidae (Ernieenor *et al*., 2021). Forty species of the genus *Dermacentor* have been reported to be distributed worldwide, and 12 species have been found in China (Guo *et al*., 2016; Nava *et al*., 2017). All species of the genus *Dermacentor* have distinct external morphological characteristics and are classified in the subgenus *Indocentor* Schulze (Ernieenor *et al*., 2020). Hosts of the genus *Dermacentor* include horses, cattle, sheep and rodents, and can transmit a variety of pathogens, including *Babesia bovis*, *B. caballi*, *B. canis*, *Theileria equi* and other protozoa; Brucella, Rickettsia, Rocky Mountain spotted fever, Anaplasma and other bacteria, as well as tick-borne encephalitis virus (Parola *et al*., 2005; Swei *et al*., 2019; Ličková *et al*., 2020; Duncan *et al*., 2021; Eisen and Paddock, 2021; Yang *et al*., 2021). In addition, species of the genus *Dermacentor* are also most frequently associated with human intra-ear infections (Mariana *et al*., 2008). Among them, *Dermacentor steini* is considered to be the most abundant member of the subgenus *Indocentor* and is mainly parasitic on wild boars in Asian countries (Wassef and Hoogstraal, 1988; Lim *et al*., 2020). *D. steini* is morphologically most similar to *D. auratus* and *D. atrosignatus* and carries pathogens (*Rickettsia* sp., *Anaplasma* sp., Lanjan virus and *Hepatozoon* species) similar to those isolated from *D. auratus* (Wassef and Hoogstraal, 1986, 1988). Most importantly, *D. steini* feeds on human skin or other tissues and is an important reservoir host and vector of zoonotic diseases, which poses a greater threat to public health safety (Vongphayloth *et al*., 2016; Lim *et al*., 2020).

The mitochondrial genome (mitogenome) is a DNA molecule typically 15–20 kb in size, with 37 genes, including 13 protein-coding genes (PCGs), 22 transfer RNA genes (tRNAs), 2 ribosomal RNA genes (rRNAs) and usually longer non-coding regions (control regions) (Wang *et al*., 2018, 2020). The mitochondrial genome is widely considered as one of the most reliable and effective genetic markers in molecular phylogenetic studies because of its maternal inheritance, high mutation rate and lack of recombination (Yang *et al*., 2022*a*, 2022*b*). Meanwhile, the mitochondrial genome has been proven to be a useful tool for understanding the evolutionary, geographic and pathogenic relationships of tick species (Burger *et al*., 2013).

Given the wide distribution of ticks of the genus *Dermacentor* and their importance as vectors of zoonotic diseases, they have an important role in the development of human and livestock production. Thus, this study reports the mitochondrial genome sequence of *D. steini* (Longyan isolate) collected in Longyan, Fujian Province, China, and compared with those of 3 other *D. steini* (Jinhua, Nangchang and Yingtan isolates) from different regions of China.

## Materials and methods

### Sample collection

Rats were captured using mousetraps in Longyan, Fujian Province, China. Adult ticks were then collected from the body surface of the captured rats (*Rattus andamanensis*) and preserved in Eppendorf (EP) tubes with 95% ethanol, and the sample number was labelled P299.

*Statement:* The capture protocol and procedures for small mammals were approved by the Animal Ethics Committee of Dali University. The approval number is MECDU-201806-11.

### DNA extraction and mitogenome sequencing

The adult ticks were taken out of the EP tubes containing 95% ethanol and immersed in sterile distilled water for 30 min to remove microorganisms from the surface, and then the ticks were cut with a sterile scalpel blade. Genomic DNA was extracted from ticks with the DNeasy Blood and Tissue Kit (Qiagen, Valencia, California, USA). The extracted DNA was used to construct Illumina PE libraries. The mitochondrial genome was then sequenced on the Illumina Novoseq 6000 platform (Winnerbio, Shanghai, China).

### Genome annotation

MitoZ 2.3 (https://doi.org/10.1101/489955) was used to assemble the mitochondrial genome. The MITOS web server was used to predict genes (Bernt *et al*., 2013), and 13 PCGs were compared and edited using the BLAST tool in NCBI. The location and length of tRNA genes were further confirmed using tRNAscan-SE (Lowe and Eddy, 1997) and ARWEN (Laslett and Canbäck, 2008). Two rRNA genes were identified based on their putative secondary structures and previously sequenced mitochondrial genome sequences. The position of the control region was confirmed based on the boundaries of neighbouring genes. The mitochondrial sequence of *D. steini* (Longyan isolate) has been deposited in GenBank under the accession number OP383032.

### Sequence analysis and phylogenetic reconstruction

Base composition and codon usage were calculated using GeneiousPrime (Kearse *et al*., 2012) and MEGA X (Kumar *et al*., 2016), respectively. Sliding window analysis was performed on the complete mitochondria of different *D. steini* isolates using DnaSP v5 (Librado and Rozas, 2009). The sliding window length was set to 100 bp with a step size of 25 bp to estimate the value of nucleotide diversity. Phylogenetic trees were constructed using Bayesian inference (BI) methods and maximum likelihood (ML) methods with *Limulus polyphemus* and *Carcinoscorpius rotundicauda* as outgroups. BI and ML analyses were performed in PhyloSuite (Zhang *et al*., 2020). For the Bayesian analysis, a total of 1 000 000 generations were run, with sampling every 1000 generations. The first 25% of the tree was burned to ensure sample independence and 4 Monte Carlo Markov chains were run. To estimate the support of the BI tree, posterior probabilities (PP) were calculated. For the ML tree, 5000 ultra-fast bootstrap replications were used to calculate branch reliability (bootstrap probability, BP). The constructed phylogenetic tree was viewed and edited with FigTree 1.4.1 (https://github.com/rambaut/figtree/). Species and accession numbers for the construction of the phylogenetic tree are shown in Supplementary Table 1.

## Results

### Mitochondrial genome characteristics of *Dermacentor steini* (Longyan isolate)

The mitochondrial genome of *D. steini* (Longyan isolate) is a circular double-stranded DNA molecule with a length of 14 785 bp. It includes 13 PCGs (*cox1-3*, *cytb*, *nad1-6*, *nad4L*, *atp6* and *atp8*), 22 tRNA genes, 2 rRNA genes (*rrnL* and *rrnS*) and 2 control regions (Table 1), which is consistent with the reported mtgenomes of other species of the genus *Dermacentor* but not with the mitochondrial genomes of some metazoan, such as in *trematodes*, *cestodes* and *Chromadorea nema-todes*, which have been found to have lost the *atp8* gene in their mitochondrial genomes (Yamasaki *et al*., 2012; Duan *et al*., 2015; Chang *et al*., 2016). Of the 37 genes, 23 genes (*cox1-3*, *nad2-3*, *nad6*, *atp6*, *atp8*, *cytb*, *trnA*, *trnC*, *trnD*, *trnE*, *trnG*, *trnI*, *trnK*, *trnM*, *trnN*, *trnR*, *trnS*_1_, *trnS*_2_, *trnT*, *trnW*) are encoded on the J-strand, and the remaining 12 genes (*nad1*, *nad4*, *nad4L*, *nad5*, *trnF*, *trnH*, *trnL*_1_, *trnL*_2_, *trnP*, *trnQ*, *trnV*, *trnY*) and 2 rRNA genes (*16S rRNA* and *12S rRNA*) are encoded on the N-strand.

**Table 1.** *Dermacentor steini* gene content, length, coding strand, initiation and stop codons of mitochondrial genomes of different isolate

Gene/regions	Strand				Positions and lengths (bp)				Initiation and stop codons			
	L.Y	N.C	J.H	Y.T	L.Y	N.C	J.H	Y.T	L.Y	N.C	J.H	Y.T
*trnM*	J	J	J	J	1–67 (67)	1–67 (67)	1–67 (67)	1–67 (67)				
*nad2*	J	J	J	J	68–1027 (960)	68–1027 (960)	68–1027 (960)	68–1027 (960)	ATT/TAA	ATT/TAA	ATT/TAA	ATT/TAA
*trnW*	J	J	J	J	1026–1087 (62)	1026–1087 (62)	1027–1086 (62)	1026–1087 (62)				
*trnY*	N	N	N	N	1092–1154 (63)	1092–1154 (63)	1092–1154 (63)	1092–1154 (63)				
*cox1*	J	J	J	J	1147–2685 (1539)	1147–2685 (1539)	1147–2685 (1539)	1147–2685 (1539)	ATT/TAA	ATT/TAA	ATT/TAA	ATT/TAA
*cox2*	J	J	J	J	2690–3362 (673)	2690–3362 (673)	2690–3362 (673)	2690–3362 (673)	ATG/T	ATG/T	ATG/T	ATG/T
*trnK*	J	J	J	J	3363–3428 (66)	3363–3427 (65)	3363–3428 (66)	3363–3428 (66)				
*trnD*	J	J	J	J	3428–3487 (60)	3429–3488 (60)	3428–3488 (61)	3427–3487 (61)				
*atp8*	J	J	J	J	3488–3646 (159)	3488–3646 (159)	3488–3646 (159)	3488–3646 (159)	ATC/TAA	ATC/TAA	ATC/TAA	ATC/TAA
*atp6*	J	J	J	J	3640–4305 (666)	3640–4305 (666)	3640–4305 (666)	3640–4305 (666)	ATG/TAA	ATG/TAG	ATG/TAG	ATG/TAA
*cox3*	J	J	J	J	4330–5108 (779)	4330–5108 (779)	4323–5100 (778)	4323–5101 (779)	ATG/TA	ATG/TA	ATG/T	ATG/TA
*trnG*	J	J	J	J	5107–5169 (63)	5108–5168 (61)	5101–5161 (61)	5102–5161 (60)				
*nad3*	J	J	J	J	5166–5507 (342)	5166–5507 (342)	5162–5500 (339)	5162–5500 (339)	ATA/TAA	ATA/TAA	ATT/TAA	ATT/TAA
*trnA*	J	J	J	J	5507–5570 (64)	5507–5569 (63)	5500–5563 (64)	5500–5563 (64)				
*trnR*	J	J	J	J	5572–5639 (68)	5572–5637 (66)	5565–5632 (68)	5565–5632 (68)				
*trnN*	J	J	J	J	5634–5699 (66)	5634–5699 (66)	5638–5691 (54)	5628–5690 (63)				
*trnS*_1_	J	J	J	J	5702–5759 (58)	5702–5759 (58)	5696–5751 (56)	5696–5756 (61)				
*trnE*	J	J	J	J	5759–5819 (61)	5758–5820 (63)	5752–5812 (61)	5752–5813 (62)				
*nad1*	N	N	N	N	5818–6756 (939)	5818–6756 (939)	5811–6749 (939)	5811–6749 (939)	ATT/TAA	ATT/TAA	ATT/TAA	ATT/TAA
*trnL*_2_	N	N	N	N	6757–6818 (62)	6757–6818 (61)	6750–6811 (62)	6750–6811 (62)				
*16S rRNA*	N	N	N	N	6819–8018 (1200)	6819–8018 (1200)	6812–8004 (1193)	6812–8004 (1193)				
*trnV*	N	N	N	N	8015–8075 (61)	8017–8077 (61)	8004–8064 (61)	8004–8064 (61)				
*12S rRNA*	N	N	N	N	8077–8788 (712)	8079–8786 (708)	8006–8775 (710)	8066–8771 (706)				
*CR1*					8789–9100 (312)	8787–9100 (314)	8776–9088 (313)	8772–9084 (313)				
*trnI*	J	J	J	J	9101–9163 (63)	9101–9163 (63)	9089–9151 (63)	9085–9147 (63)				
*trnQ*	N	N	N	N	9172–9237 (66)	9172–9237 (66)	9160–9225 (66)	9156–9221 (66)				
*trnF*	N	N	N	N	9264–9327 (64)	9264–9327 (64)	9252–9315 (64)	9248–9309 (62)				
*nad5*	N	N	N	N	9327–10 985 (1659)	9327–10 985 (1659)	9315–10 973 (1659)	9311–10 969 (1659)	ATT/TAA	ATT/TAA	ATT/TAA	ATT/TAA
*trnH*	N	N	N	N	10 986–11 048 (63)	10 986–11 048 (63)	10 974–11 036 (63)	10 970–11 032 (63)				
*nad4*	N	N	N	N	11 049–12 363 (1315)	11 049–12 363 (1315)	11 037–12 351 (1315)	11 033–12 347 (1315)	ATG/T	ATG/T	ATG/T	ATG/T
*nad4L*	N	N	N	N	12 357–12 632 (276)	12 357–12 632 (276)	12 345–12 620 (276)	12 341–12 616 (276)	ATG/TAA	ATG/TAA	ATG/TAA	ATG/TAA
*trnT*	J	J	J	J	12 634–12 697 (64)	12 635–12 698 (64)	12 623–12 683 (61)	12 619–12 681 (64)				
*trnP*	N	N	N	N	12 695–12 757 (63)	12 696–12 756 (61)	12 684–12 744 (61)	12 680–12 740 (61)				
*nad6*	J	J	J	J	12 759–13 193 (435)	12 759–13 193 (435)	12 732–13 181 (450)	12 728–13 177 (450)	ATT/TAA	ATT/TAA	ATA/TAA	ATA/TAA
*cytb*	J	J	J	J	13 208–14 290 (1083)	13 208–14 290 (1083)	13 196–14 278 (1083)	13 190–14 272 (1083)	ATG/TAG	ATG/TAG	ATG/TAG	ATG/TAG
*trnS*_2_	J	J	J	J	14 289–14 355 (67)	14 290–14 355 (66)	14 277–14 344 (68)	14 272–14 337 (66)				
*trnL*_1_	N	N	N	N	14 355–14 417 (63)	14 355–14 417 (63)	14 343–14 405 (63)	14 337–14 399 (63)				
*CR2*					14 418–14 724 (307)	14 418–14 725 (308)	14 406–14 713 (308)	14 400–14 706 (307)				
*trnC*	J	J	J	J	14 725–14 781 (57)	14 725–14 781 (57)	14 714–14 767 (54)	14 707–14 767 (61)				

L.Y, Longyan; N.C, Nanchang; J.H, Jinhua; Y.T, Yingtan

The mitochondrial genome of *D. steini* (Longyan isolate) is relatively compact with 12 gene intergenic regions and 16 gene overlap regions (Table 1), with total lengths of 90 and 52 bp, respectively. The intergenic region occurred at 23 gene junctions. The longest intergenic sequence (26 bp) was found between *trnQ* and *trnF*, and the shortest intergenic sequence (1 bp) was found between *trnA* and *trnR*, *trnV* and *12S rRNA*, *nad4L* and *trnT,* and *trnP* and *nad6*. Overlap regions occurred at 29 gene junctions. The longest overlap sequence, 8 bp, was found between *trnY* and *cox1*. The shortest overlap region, 1 bp, was found between *nad3* and *trnA*, *trnS*_1_ and *trnE*, *trnF* and *nad5,* and *trnS*_2_ and *trnL*_1_. Among them, the 7 bp overlap between the *nad4* and *nad4L* genes has also been widely reported in the mitochondrial genome of insects (Wang and Tang, 2017).

**Fig. 1.**
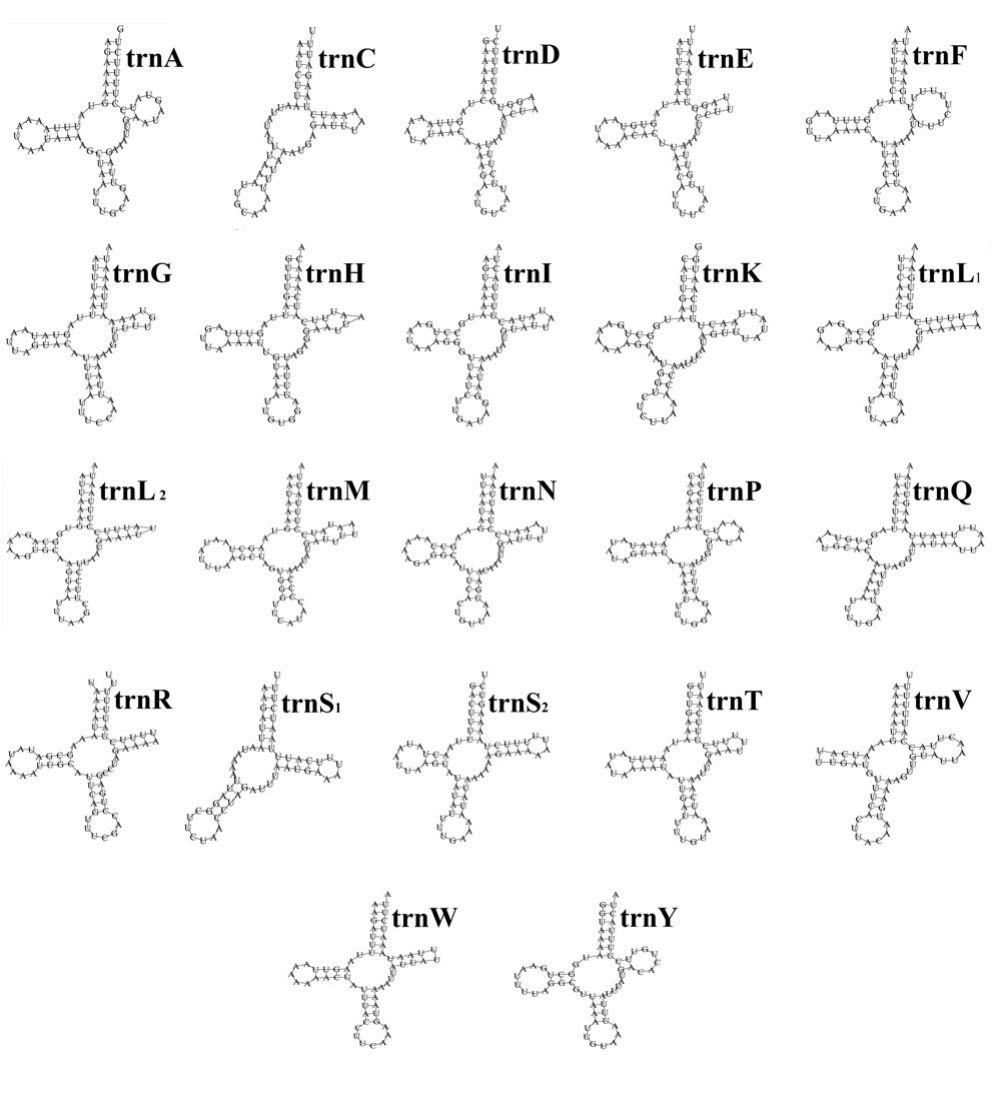
*Dermacentor steini* (Longyan isolate) tRNA gene putative secondary structure.

The typical secondary structure of tRNA genes is a cloverleaf structure with 4 arms: amino acid acceptor arm (AA-arm), DHC arm (D-arm), anticodon arm (AC-arm) and TΨC arm (T-arm) (Yuan *et al*., 2010). The 22 tRNA genes of *D. steini* (Longyan isolate) have a total length of 1391 bp. The length of individual tRNA genes ranged from 57 bp (*trnC*) to 68 bp (*trnR*), and their secondary structures are shown in Fig. 1. Except for *trnC* and *trnS*_1_, which lacked the D-arm, the other 20 tRNA genes were able to form a typical cloverleaf structure. Among these tRNA genes, 14 tRNA genes are encoded in the J-strand and the other 8 tRNA genes are encoded in the N-strand. Besides the typical Waston–Crick pairings (A–U, G–C), 12 non-canonical pairings, 9 G–U pairings and 3 U–U mismatches occurred.

### Comparative analysis between mitochondrial genomes of *D. steini* (Longyan, Nanchang, Jinhua and Yingtan isolates)

**Fig. 2.**
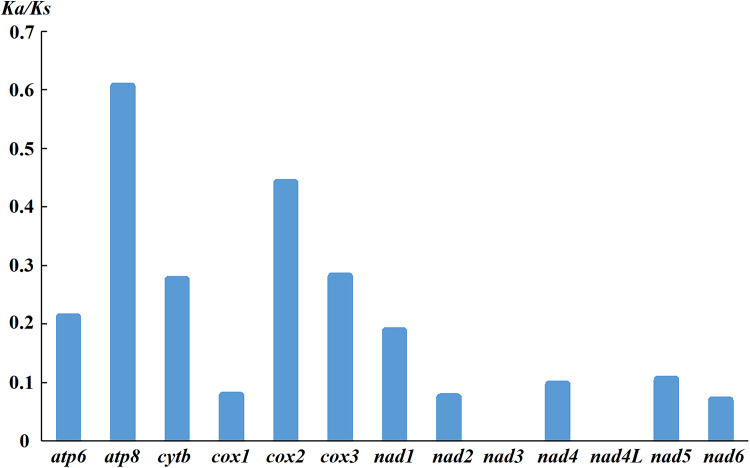
Selection pressure analysis for different isolates in *Dermacentor steini*.

The mitochondrial genomes of *D. steini* isolated from Longyan and Nanchang were of the same length, 12 and 18 bp longer than those of Jinhua and Yingtan isolates, respectively. The 37 genes and 2 non-coding regions of the *D. steini* from Longyan, Nanchang, Jinhua and Yingtan isolate mitochondrial genomes were identical or similar in length, and the overall variation between sequences was 0.76%. From Table 2, the nucleotide composition of the mitochondrial genomes of the 4 isolates (Longyan, Nanchang, Jinhua and Yingtan) showed a distinct AT preference. All PCGs used the typical ATN as the initiation codon and no rare initiation codons (GTG and TTG) were present; of which; ATT and ATG were used up to 5 and 6 times, respectively. With the use of stop codons, most PCGs ended with the complete stop codon TAA. The truncated stop codon T/TA was found in the *cox2*, *cox3* and *nad4* genes of 4 isolates (Longyan, Nanchang, Jinhua and Yingtan). Figure 2 shows an analysis of the selection pressure of each protein-coding gene, which revealed that the ratio of non-synonymous mutation to synonymous mutation (Ka/Ks) for each protein-coding gene was less than 1, and the ratio of *nad3* and *nad4L* was 0.

**Table 2.** *Dermacentor steini* variation in nucleotide and predicted amino acid sequences of the mitochondrial genome of different isolates

*D. steini*	Nucleotide sequence length				Nucleotide variance (%)	Number of amino acids				Amino acids variance (%)
	Longyan	Jinhua	Nangchang	Yingtan		Longyan	Jinhua	Nanchang	Yingtan	
Mitogenome	14 785	14 773	14 785	14 767	0.76	–	–	–	–	–
AT%	76.9	76.9	76.9	76.8	–	–	–	–	–	–
*cox1*	1539	1539	1539	1539	0.97	512	512	512	512	0.20
*cox2*	673	673	673	673	0.74	224	224	224	224	0.13
*cox3*	779	778	779	779	0.90	259	259	259	259	0.77
*nad1*	939	939	939	939	0.53	312	312	312	312	0.32
*nad2*	960	960	960	960	0.94	319	319	319	319	1.56
*nad3*	342	339	342	339	0.00	113	112	113	112	0.88
*nad4*	1315	1315	1315	1315	0.61	438	438	438	438	0.46
*nad4L*	276	276	276	276	0.36	91	91	91	91	0.00
*nad5*	1659	1659	1659	1659	0.42	552	552	552	552	0.36
*nad6*	435	450	435	450	1.11	144	149	144	149	0.67
*atp6*	666	666	666	666	0.45	221	221	221	221	0.00
*atp8*	159	159	159	159	1.89	53	53	53	53	3.85
*cytb*	1083	1083	1083	1083	0.55	360	360	360	360	0.83
*rrnL*	1200	1193	1200	1193	1.00	–	–	–	–	–
*rrnS*	712	710	708	706	1.54	–	–	–	–	–

The nucleotide and amino acid variations of the 13 PCGs in Longyan, Nanchang, Jinhua and Yingtan isolates are shown in Table 2. The highest variation in nucleotides between homologous genes was found for *atp8* (1.89%), and with lowest variation gene was *nad3* (0.00%). For amino acid sequences, the lowest variation genes were nad4 and *atp6* (0.00%), and the *atp8* gene had the highest variation (5.66%). These results suggest that the *atp8* gene was the most variable. The 2 rRNAs (*rrnL* and rr*nS*) of the Longyan, Nanchang, Jinhua and Yingtan isolates were similar in length to other ticks (Burger *et al*., 2014; Williams-Newkirk *et al*., 2015). *rrnL* was located between *trnL* and *trnV* with a 1.00% nucleotide variation; *rrnS* was located between *trnV* and NCR1 with a nucleotide variation of 1.54%.

**Fig. 3.**
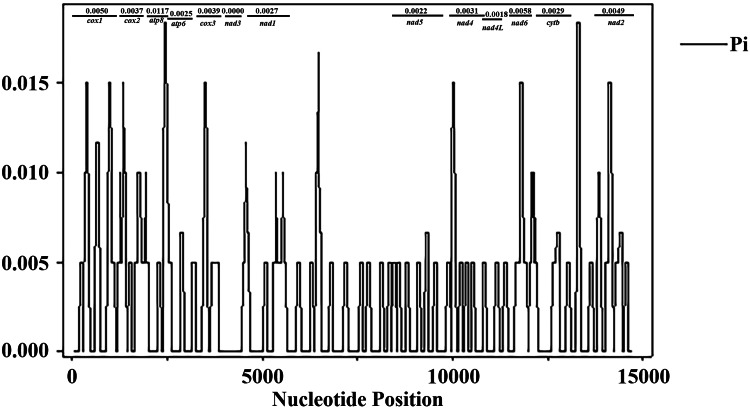
Sliding window analysis of the complete mitochondrial genomes of *Dermacentor steini* different isolates. The black line indicates the nucleotide diversity values (Pi) in the sliding window analysis with a window size of 100 bp and a step size of 25; the gene boundaries are indicated by the variation rate of each gene.

The nucleotide diversity (Pi) of the 13 PCGs of *D. steini* (Longyan, Nanchang, Jinhua and Yingtan isolates) was analysed using DnaSP (Fig. 3). It was found that the nucleotide variation of *D. steini* within and between genes was low, in the range of 0.0000–0.0117. According to the window analysis, the most variable sequences were *atp8* (0.0117), *nad6* (0.0058), *cox1* (0.0050) and *nad2* (0.0049), while the most conserved sequences were *nad3* (0.000).

### Rearrangement and phylogenetic analysis of the mitochondrial genome of *D. steini* (Longyan, Nanchang, Jinhua and Yingtan isolates)

**Fig. 4.**
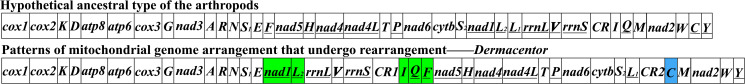
Mitochondrial genome rearrangement evolution in the genus *Dermacentor*. Green indicates translocations, blue indicates translocations and inversions.

**Fig. 5.**
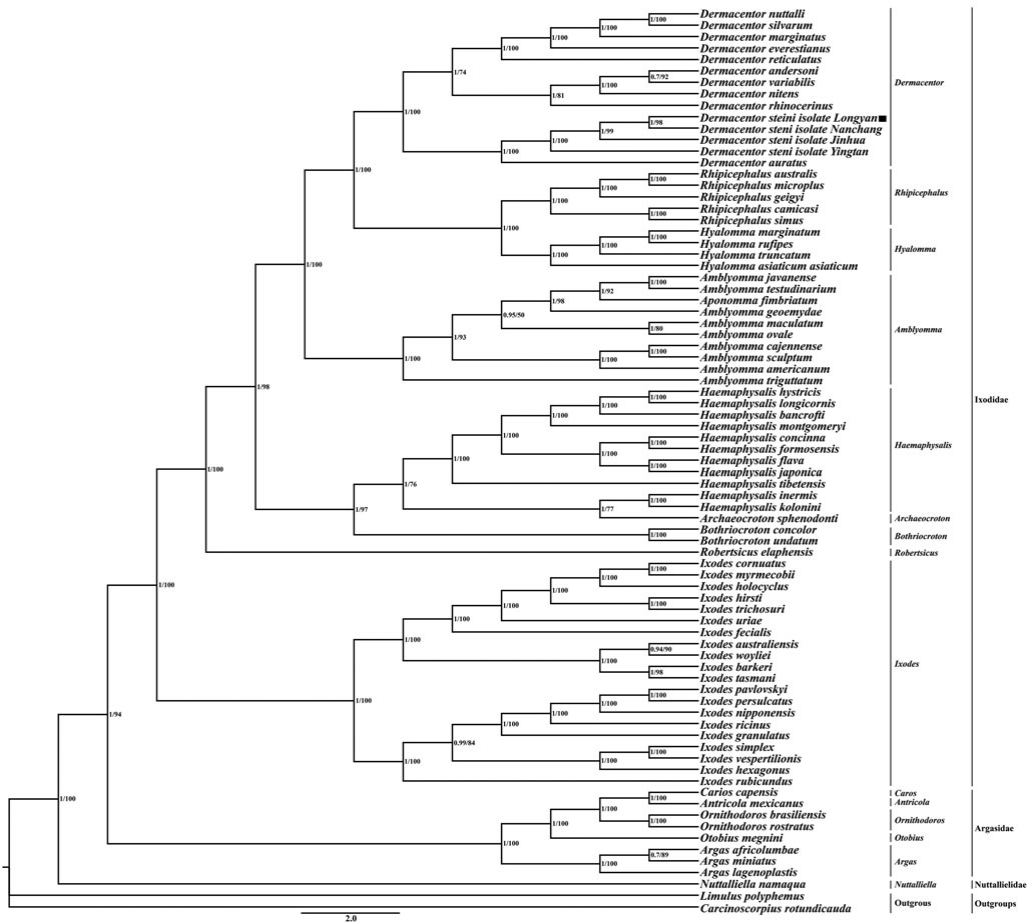
Phylogenetic analysis based on the nucleotide sequences of the 13 PCGs in the mitogenome. The numbers beside the nodes are posterior probabilities (BI) and bootstrap (ML) indicates the species in this study.

Although the mitochondrial genome evolves at a rapid rate, the order of the 37 genes is very conservative, and the rearrangement characteristics of mitochondrial genes are generally used to resolve controversial phylogenetic questions (Babbucci *et al*., 2014). Compared to the mitochondrial genome the arrangement pattern of arthropod hypothetical ancestors, smaller rearrangements occurred in the mitochondrial genomes of *D. steini* (Longyan, Nanchang, Jinhua and Yingtan isolates) and other species of the genus *Dermacentor* (Fig. 4). Rearrangements occurred mainly in 3 regions, namely, *nad1*-*trnL*_2_, *trnI*-*trnQ*-*trnF*, underwent translocations, as well as *trnC* underwent translocations and inversions, and all rearrangements occurred in the vicinity of control regions and tRNA genes. Phylogenetic trees were constructed using the maximum likelihood and Bayesian methods based on 13 PCGs with *L. polyphemus* (NC003057) and *C. rotundicauda* (JX437074) as outgroups (Fig. 5). The topology of the phylogenetic tree formed by the 2 methods was almost identical, except for slight differences in the support rates of branch nodes: the support rate of the Bayesian tree (BI) was generally higher than that of the maximum likelihood tree (ML), with the majority of nodes having a support rate of 1; while the support rates of the maximum likelihood tree (ML) were all in the range of 80–100, except for 3 nodes with a support rate of less than 80. The phylogenetic tree shows that the family Ixodidae is a monophyletic group, except for the genus *Archaeocroton*, in which all other genera are clustered separately into a single group. *D. steini* isolated from Longyan and Nanchang clustered preferentially together and then formed sister branches with high node support (BP > 95, PP > 0.95) to *D. steini* isolated from Jinhua and Yingtan. The present phylogenetic tree results show that the genus *Dermacentor* is most closely related to 2 genera (*Rhipicentor* and *Hyalomma*) in the family Ixodidae (BP > 95, PP > 0.95).

## Discussion

Most species of the genus *Dermacentor* are 3-host ticks, with larval and nymph ticks mainly parasitizing small mammals and adult ticks mainly parasitizing cattle, sheep and wild medium and large mammals, and are capable of transmitting a variety of diseases to humans and animals (Hajdušek *et al*., 2013; Ernieenor *et al*., 2020). Molecular biology studies are essential for the surveillance, prevention and control of tick-borne diseases.

In this study, the complete mitochondrial genome of *D. steini* (Longyan isolate) was sequenced and compared with the Nanchang, Jinhua and Yingtan isolates. The mitochondrial genome of *D. steini* (Longyan, Nanchang, Jinhua and Yingtan isolates) was found to be shorter than that of most species of the genus *Dermacentor*. The size of the mitochondrial genome varied among species, mainly due to the differences in the number and length of non-coding regions (Mo *et al*., 2019). The nucleotide composition of *D. steini* (Longyan, Nanchang, Jinhua and Yingtan isolates) showed a distinct AT preference, ranging from 76.8% to 76.9%, which is within the reported range of the *Dermacentor* genus ticks (Xin *et al*., 2018). It has been suggested that the higher AT content than GC content is a common derivative of arthropod mitochondrial genomes (Ikemura, 1985; Simon *et al*., 1994). All 37 mitochondrial genes of *D. steini* (Longyan, Nanchang, Jinhua and Yingtan isolates) were non-homogeneously distributed. Of the 23 genes located in the J-strand, the remaining genes were in the N-strand. In general, mitochondrial genes located on the J-strand are more susceptible to hydrolysis and oxidation (Brown *et al*., 1982). Among the 13 PCGs of *D. steini* (Longyan, Nanchang, Jinhua and Yingtan isolates), 4 genes (*nad1*, *nad4*, *nad4L*, *nad5*) were located on the N-strand, indicating that these 4 genes have relative stability.

The initiation codon and stop codon differ between species, and variations in the genetic code can be used as specific markers of the mitochondrial genome (Murrell *et al*., 2003). For example, ATN is considered to be the most common initiation codon in Insecta, while AGA and AGG are the most common stop codons in vertebrate mitochondrial genomes (Wolstenholme, 1992; Wang *et al*., 2019). The most common initiation and stop codons of *D. steini* (Longyan, Nanchang, Jinhua and Yingtan isolates) are ATG and TAA, respectively, which are also found in the mitochondrial genomes of other ticks (Liu *et al*., 2018). Three PCGs (*cox2*, *cox3* and *nad4*) have incomplete stop codons, and those incomplete stop codons can be modified by post-transcriptional polyadenylation to eventually form complete stop codons (Donath *et al*., 2019). The mitochondrial protein-encoding genes have been conserved (Chen *et al*., 2012). The Ka/Ks of the 13 PCGs of *D. steini* (Longyan, Nanchang, Jinhua and Yingtan isolates) were all less than 1, indicating that these 13 PCGs were subject to purifying selection and did not undergo rapid evolution. The Ka/Ks of *nad3* and *nad4L* were 0, further indicating that these 2 genes are highly conserved.

As the mitochondrial tRNA genes are approximately the same size, in comparison to nuclear tRNA genes, mitochondrial tRNA genes have a higher evolution rate (Saccone *et al*., 1999). The *trnS*_1_ gene lacks the D-arm and fails to form a typical cloverleaf secondary structure, which is considered to be an ancestral characteristic of metazoan (Lowe and Chan, 2016). The 22 tRNA genes of *D. steini* (Longyan isolate) also have the *trnC* gene lacking the D-arm. Dermauw *et al*. considered the *trnC* gene lacking D-arm as a common ancestral characteristic of Mesostigmata (Acari: Parasitiformes) species (Dermauw *et al*., 2010), but it has not been confirmed in the mitochondrial genome of ticks. The *D. steini* (Longyan isolate) tRNA gene underwent 9 G–U pairings and 3 U–U mismatches during the folding process. Compared with bacterial and cytoplasmic tRNA genes, mitochondrial tRNA genes contain more G–U pairing and mismatching, which is an important factor that makes mitochondrial tRNA genes less stable than cytoplasmic tRNA genes (Helm *et al*., 2000). The G–U pairing and mismatches that occur on mitochondrial tRNA genes are highly conserved in evolution or involved in the formation of higher structures that can be corrected by RNA editing, and their normal transport function is not affected, which is important for maintaining the stability and specificity of tRNA genes (Kunzmann *et al*., 1998; Helm *et al*., 2000). It has been thought that mismatches in tRNA genes may be linked to species evolution (Watanabe *et al*., 1994). Hence, whether the base mismatches occurring in the mitochondrial tRNA gene of *D. steini* (Longyan isolate) are related to its evolution needs to be further explored.

Geographic location is generally considered to be one of the factors influencing variation in the mitochondrial genome (Burnard and Shao, 2019). The overall variation among the mitochondrial genome sequences of *D. steini* (Longyan, Nanchang, Jinhua and Yingtan isolates) was 0.76%, which was less than 1%. It suggests that the variation in the mitochondrial genome of *D. steini* may be less affected by geographical location; it may also be due to the proximity (<1000 km) between the 4 locations (Longyan, Nanchang, Jinhua and Yingtan), which does not reflect the intra-species variation. The nucleotide and amino acid sequences of homologous genes as well as nucleotide diversity analysis showed that *atp8* was the least conserved gene, which is consistent with the results of other tick species (Guo *et al*., 2016). It further suggests that the *atp8* gene can be used as an alternative genetic marker for studying *D. steini* from different host body surfaces and different biogeographies.

The altered gene order of the mitochondrial genome is a well-known specific molecular marker that contains important phylogenetic information (Boore, 1999; Boore and Fuerstenberg, 2008). The study of mitochondrial genome rearrangement events is one of the hotspots for studying the evolutionary process of species. The mitochondrial genome arrangement is generally considered to be highly conserved at lower taxonomic levels (families and genera) and rearrangements occur much more frequently for short fragments than for long fragments (Dowton *et al*., 2009; Yuan *et al.* 2010). The mitochondrial genome rearrangements of species within the genus *Dermacentor* are relatively simple, not occurring in some very conserved regions (*rrnL*-*trnV*-*rrnS*), but all in 3 regions (*nad1*-*trnL*_2_, *trnI*-*trnQ*-*trnF* and *trnC*). These 3 regions are located in the vicinity of the control region and tRNA genes. It has been demonstrated in the mitochondrial genomes of sucking lice, insects and mites that the control region and the vicinity of tRNA genes are the hotspot regions in which rearrangements occur (Wei *et al*., 2009, 2010; Dong *et al*., 2021). It has been suggested that replication of the control region is responsible for the accelerated frequency of mitochondrial gene rearrangements, as in Thrips and Corrodentia (Shao and Barker, 2003; Shao *et al*., 2005; Yan *et al*., 2012). It remains to be verified whether the accelerated frequency of mitochondrial genome rearrangements in ticks is related to the replication of control regions. The BI and ML trees indicate that the family Ixodidae is a monophyletic group and the phylogenetic relationships within the family are relatively clear. *D. steini* isolated from Longyan clustered with 3 other isolates (Nanchang, Jinhua and Yingtan), and then formed a sister branch with *D. auratus* with high node support. It indicates that among the genus *Dermacentor*, *D. steini* is most closely related to *D. auratus*. It may be related to the similarity of these 2 species in morphology and in the pathogens they carry. Two phylogenetic trees showed that the genus *Dermacentor* is most closely related to 2 genera (*Rhipicentor* and *Hyalomma*), which is consistent with previous studies (Burnard and Shao, 2019; Uribe *et al*., 2020; Kelava *et al*., 2021). The analysis of the affinities of the genus *Dermacentor* facilitates the subsequent understanding of the phylogenetic analysis of species in the genus *Dermacentor*.

## Conclusion

In this study, the complete mitochondrial genome of *D. steini* (Longyan isolate) parasitized on *R. andamanensis* was sequenced and compared with Nanchang, Jinhua and Yingtan isolates. The results showed that the mitochondrial genomes of *D. steini* different isolates were similar in length and the overall variation between sequences was less than 1%; the *atp8* gene may serve as an alternative genetic marker for studying *D. steini* species from different hosts and biogeography; the mitochondrial genome underwent minor rearrangements. Phylogenetic analyses showed that the genus *Dermacentor* is most closely related to 2 genera (*Rhipicentor* and *Hyalomma*) in the family Ixodidae. The results enrich the mitochondrial genomic data of species in the genus *Dermacentor* and have important implications for the phylogeographic classification and molecular evolution of ticks.

## Data Availability

The complete mitochondrial genome sequence of *D. steini* is available at the National Center for Biotechnology Information (NCBI) at [https://www.ncbi.nlm.nih.gov/] under accession number OP383032.

## Supplementary material

For supplementary material accompanying this paper visit https://doi.org/10.1017/S0031182022001639. click here to view supplementary material
