# Supplementary material for: Comparative analysis of the mitochondrial genome of Dermacentor steini from different regions in China
Source: Parasitology. 2022 Dec 9;150(2):195–205. doi: 10.1017/S0031182022001639 (PMC10090625; doi:10.1017/S0031182022001639)
Supplement: Supplementary file 1 [file S0031182022001639sup001.docx]

**Supplementary Table 1.** Species information for constructing phylogenetic trees.

| Family | Genus | Species | GenBank no. | Length(bp) |
| --- | --- | --- | --- | --- |
| Ixodidae | *Dermacentor* | *Dermacentor nuttalli* | NC028528 | 15086 |
|  |  | *Dermacentor silvarum* | NC026552 | 14945 |
|  |  | *Dermacentor marginatus* | MK905212 | 15067 |
|  |  | *Dermacentor everestianus* | NC042764 | 15191 |
|  |  | *Dermacentor reticulatus* | MT478096 | 14806 |
|  |  | *Dermacentor andersoni* | NC061057 | 14806 |
|  |  | *Dermacentor variabilis* | NC061217 | 14837 |
|  |  | *Dermacentor nitens* | NC023349 | 14839 |
|  |  | *Dermacentor rhinocerinus* | KY457526 | 14708 |
|  |  | *Dermacentor steini isolate Longyan* | OP383032 | 14785 |
|  |  | *Dermacentor steini isolate Nanchang* | OM368300 | 14785 |
|  |  | *Dermacentor steini isolate Jinhua* | OM368299 | 14773 |
|  |  | *Dermacentor steini isolate Yingtan* | OM368302 | 14767 |
|  |  | *Dermacentor auratus* | NC059724 | 14766 |
|  | *Rhipicephalus* | *Rhipicephalus australis* | NC023348 | 14891 |
|  |  | *Rhipicephalus microplus* | NC023335 | 14905 |
|  |  | *Rhipicephalus geigyi* | NC023350 | 14948 |
|  |  | *Rhipicephalus camicasi* | NC061616 | 14725 |
|  |  | *Rhipicephalus simus* | KY457542 | 14721 |
|  | *Hyalomma* | *Hyalomma marginatum* | MW366632 | 14765 |
|  |  | *Hyalomma rufipes* | KY457528 | 14748 |
|  |  | *Hyalomma truncatum* | KY457529 | 14731 |
|  |  | *Hyalomma asiaticum asiaticum* | MF101817 | 14720 |
|  | *Amblyomma* | *Amblyomma javanense* | NC043872 | 14780 |
|  |  | *Amblyomma testudinarium* | MT029329 | 14760 |
|  |  | *Aponomma fimbriatum* | NC017759 | 14705 |
|  |  | *Amblyomma geoemydae* | MK814531 | 14780 |
|  |  | *Amblyomma maculatum* | MW719251 | 14803 |
|  |  | *Amblyomma ovale* | NC050255 | 14760 |
|  |  | *Amblyomma cajennense* | NV020333 | 14780 |
|  |  | *Amblyomma sculptum* | NC032369 | 14780 |
|  |  | *Amblyomma americanum* | NC027609 | 14709 |
|  |  | *Amblyomma triguttatum* | NC005963 | 14740 |
|  | *Haemaphysalis* | *Haemaphysalis hystricis* | NC09765 | 14716 |
|  |  | *Haemaphysalis longicornis* | NC037493 | 14718 |
|  |  | *Haemaphysalis bancrofti* | NC041076 | 14673 |
|  |  | *Haemaphysalis montgomeryi* | MW751861 | 14681 |
|  |  | *Haemaphysalis concinna* | NC034785 | 14675 |
|  |  | *Haemaphysalis formosensis* | NC020334 | 14676 |
|  |  | *Haemaphysalis flava* | MG604958 | 14689 |
|  |  | *Haemaphysalis japonica* | NC037246 | 14685 |
|  |  | *Haemaphysalis tibetensis* | OM049539 | 14714 |
|  |  | *Haemaphysalis inermis* | NC020335 | 14846 |
|  |  | *Haemaphysalis kolonini* | MZ054209 | 14948 |
|  | *Archaeocroton* | *Archaeocroton sphenodonti* | NC017745 | 14772 |
|  | *Bothriocroton* | *Bothriocroton concolor* | NC017756 | 10809 |
|  |  | *Bothriocroton undatum* | NC017757 | 14769 |
|  | *Robertsicus* | *Robertsicus elaphensis* | NC017758 | 14627 |
|  | *Ixodes* | *Ixodes cornuatus* | NC062630 | 14985 |
|  |  | *Ixodes myrmecobii* | NC062632 | 14995 |
|  |  | *Ixodes holocyclus* | NC005293 | 15007 |
|  |  | *Ixodes hirsti* | NC062631 | 15040 |
|  |  | *Ixodes trichosuri* | NC062633 | 15001 |
|  |  | *Ixodes uriae* | NC006078 | 15053 |
|  |  | *Ixodes fecialis* | NC062628 | 15256 |
|  |  | *Ixodes australiensis* | NC062625 | 15217 |
|  |  | *Ixodes woyliei* | NC062627 | 15062 |
|  |  | *Ixodes barkeri* | OM302450 | 15259 |
|  |  | *Ixodes tasmani* | NC041086 | 15227 |
|  |  | *Ixodes pavlovskyi* | NC023831 | 14575 |
|  |  | *Ixodes persulcatus* | NC004370 | 14539 |
|  |  | *Ixodes nipponensis* | NC058248 | 14505 |
|  |  | *Ixodes ricinus* | JN248424 | 14566 |
|  |  | *Ixodes granulatus* | OL800705 | 14540 |
|  |  | *Ixodes simplex* | KY457531 | 14550 |
|  |  | *Ixodes vespertilionis* | MW411447 | 14559 |
|  |  | *Ixodes hexagonus* | NC002010 | 14539 |
|  |  | *Ixodes rubicundus* | KY457530 | 14565 |
| Argasidae | *Carios* | *Carios capensis* | AB075935 | 14418 |
|  | *Antricola* | *Antricola mexicanus* | KC769591 | 14415 |
|  | *Ornithodoros* | *Ornithodoros brasiliensis* | KC769593 | 14489 |
|  |  | *Ornithodoros rostratus* | KC769592 | 14452 |
|  |  | *Otobius megnini* | KC769589 | 14430 |
|  | *Argas* | *Argas africolumbae* | JQ665720 | 14440 |
|  |  | *Argas miniatus* | KC769590 | 14416 |
|  |  | *Argas lagenoplastis* | KC769587 | 14478 |
| Nuttalliellidae | *Nuttalliella* | *Nuttalliella namaqua* | JQ665719 | 14425 |
